# Nodding syndrome and epilepsy in onchocerciasis endemic regions: comparing preliminary observations from South Sudan and the Democratic Republic of the Congo with data from Uganda

**DOI:** 10.1186/s13104-016-1993-7

**Published:** 2016-03-22

**Authors:** Robert Colebunders, Adam Hendy, John L. Mokili, Joseph Francis Wamala, Joice Kaducu, Lucia Kur, Floribert Tepage, Michel Mandro, Gisele Mucinya, Germain Mambandu, Michel Yendema Komba, Jean Louis Lumaliza, Marieke van Oijen, Anne Laudisoit

**Affiliations:** Global Health Institute, Gouverneur Kinsbergen Centrum, University of Antwerp, Doornstraat 331, 2610 Antwerp, Belgium; Department of Clinical Sciences, Institute of Tropical Medicine, Antwerp, Belgium; Department of Biomedical Sciences, Institute of Tropical Medicine, Antwerp, Belgium; Biology Department, San Diego State University, San Diego, USA; Ministry of Health, Kampala, Uganda; Faculty of Medicine, Gulu University, Gulu, Uganda; Department of Neglected Tropical Diseases, Ministry of Health, Juba, Republic of South Sudan; National Onchocercosis Control Program, Ministry of Health, Kisangani, Democratic Republic of the Congo; Provincial Health Division Ituri, Ministry of Health, Bunia, Democratic Republic of the Congo; Medical Doctor Bunia, Bunia, Province Orientale Democratic Republic of the Congo; Provincial Ministry of Public Health, Kisangani, Province Orientale Democratic Republic of the Congo; Biodiversity Surveillance Center, Kisangani University, Kisangani, Democratic Republic of the Congo; General Reference Hospital of Dingila, Dingila, Democratic Republic of the Congo; Department of Neurology, Erasmus Medical Center, Rotterdam, The Netherlands; Institute of Integrative Ecology, School of Biological Sciences, University of Liverpool, Liverpool, UK

**Keywords:** Epilepsy, Nodding syndrome, Africa, *Onchocerca*, Blackflies, *Simulium*

## Abstract

**Background:**

Nodding syndrome (NS) is an epilepsy disorder occurring in children in South Sudan, northern Uganda and Tanzania. The etiology of NS is unknown, but epidemiological studies demonstrate an association between NS and onchocerciasis.

**Methods:**

Between November 2013 and July 2015 we visited onchocerciasis endemic regions in South Sudan, Uganda, and the Democratic Republic of the Congo (DRC) to assess the epilepsy situation. In South Sudan we interviewed patients and affected families, health officials, colleagues and healthcare workers, and performed a small household survey to estimate the epilepsy prevalence in the village of Mvolo, Western Equatoria State. Most information from Uganda was collected through discussions with colleagues and a review of published literature and reports. In the Bas-Uélé district of the DRC, we visited the villages of Liguga, Titule and Dingila, interviewed patients with epilepsy and family members and conducted a preliminary entomological assessment.

**Results:**

In South Sudan there is an ongoing NS and epilepsy epidemic in the Western Equatoria state that started around 1990. A survey of 22 households in Mvolo revealed that 28 out of 168 (16.7 %) children suffered from NS or another form of epilepsy. Thirteen (59 %) households had at least one child, and nine (41 %) households at least two children with NS or another form of epilepsy. In northern Uganda, an NS and epilepsy epidemic started around 2000. The occurrence of new NS cases has been in decline since 2008 and no new NS cases were officially reported in 2013. The decline in NS cases coincided with the bi-annual distribution of ivermectin and the treatment of blackfly-breeding rivers with larvicides. In Bas-Uélé district in the DRC, epilepsy appears to be endemic with cases clustered in villages close to blackfly-infested, rapid-flowing rivers. The majority of epilepsy cases in Liguga, Dingila and Titule presented with generalized (tonic–clonic) seizures without nodding, but with mental retardation. In Titule, an epilepsy prevalence of 2.3 % was documented. The only anthropophilic species of blackfly collected in the region belonged to the *Simulium damnosum* complex.

**Conclusion:**

Blackflies may play a key role in the transmission of an etiological agent that either directly or indirectly cause, not only NS, but also other forms of epilepsy in onchocerciasis endemic regions.

## Background

Nodding syndrome (NS) is an epilepsy disorder occurring in children in onchocerciasis endemic regions of South Sudan, Uganda and Tanzania [1]. NS is characterized by episodes of head nodding, a form of atonic epileptic seizure [2]. As the condition progresses individuals may develop other types of seizure and experience developmental retardation and growth stunting [1]. The head nodding seizures are often cited as being triggered by eating local foods [1, 3]. NS has severe socioeconomic implications and like other forms of epilepsy, it is associated with social stigma. Many children have died as a result of uncontrolled seizures that have led to drowning or burning [1]. The pathogenesis of NS is currently unknown, and although various infectious, toxic, nutritional, psychosocial and genetic causes have been proposed and investigated, none have been confirmed [1, 4, 5]. However, it is known that NS only occurs in onchocerciasis endemic regions and epidemiological studies indicate an association between NS and onchocerciasis [1, 6]. A meta-analysis of African data supports the hypothesis that a high intensity of *Onchocerca volvulus* infection is associated with epilepsy [7, 8].

This paper considers the current status of NS and epilepsy in three onchocerciasis endemic regions of South Sudan, Uganda, and the Democratic Republic of the Congo (DRC). It highlights gaps in current knowledge and proposes new approaches to studying the epidemiology of this neglected tropical condition.

## Methods

Between November 2013 and July 2015, we visited three onchocerciasis endemic regions, one in South-Sudan (Western Equatoria State), one in northern Uganda (Gulu, Kitgum, Pader and Lamwo districts), and one in the DRC (Orientale Province), to assess the NS and epilepsy situation. We used the 2012 World Health Organization (WHO) suspected case definition of NS [9] and a history of unprovoked seizures as a definition for epilepsy [10].

In South Sudan, we visited the towns and villages of Mundri, Yeri and Mvolo (N6.060121, E29.952274), and Lui Hospital between 19th and 26th November 2013. In each location we interviewed patients and affected families, health officials and healthcare workers. In Mvolo (N6.060121, E29.952274), we performed a small household survey to estimate the prevalence of epilepsy in the village. The survey was carried out over 2 days by a team consisting of R. Colebunders, M. van Oyen (neurologist), S. Komoyngi (chairman of a local NGO to support patients with nodding syndrome) and J. Podi (community health worker in Mvolo).

The northern Ugandan towns and trading centers of Gulu, Kitgum, and Padwat Central were visited on 27th November 2013. On 26th July 2015 we visited the NS treatment facility “Hope for Humans” in Odek, and on 30th July, Tumangu, a village in Kitgum district where 105 NS cases were recorded during a 2012 Ministry of Health survey. Most information about Uganda was collected through discussions with colleagues and in a review of published literature and reports.

In March 2014, an initial exploratory mission to the Bas Uélé district of the DRC included visits to Dingila (N3.68336, E26.03611), Titule (N3.28150, E25.52894) and Liguga (N3.40253, E25.02586). This was followed by a second mission to Dingila and Titule between 8th and 19th June 2014. Dingila is located along the Uélé river and Titule is located along the Bima river which is a major tributary of the Uélé. Liguga is situated at the confluence of the two rivers. The main socio-economic activities in the region are agriculture and fishing. In all three towns we interviewed patients with epilepsy and family members. In Titule, a preliminary entomological assessment was also conducted to identify the predominant human-biting blackfly species.

### Human subjects

Approval of study protocols was obtained from the Sudanese Ministry of Health ethics committee, from the Ministry of Health of the Orientale Province, and the ethics committee of the University of Kisangani, DRC. Informed consent was obtained from patients and families who were interviewed. In Uganda we did not perform any interviews with patients and families, information was obtained through discussions with colleagues and a review of published literature and reports.

### Entomological studies

Authorization to collect, transport and ship biological material was granted by the Biodiversity Monitoring Center (CSB, Centre de Surveillance de la Biodiversité), at the University of Kisangani, DRC.

## Results

### South Sudan

In the villages of Mundri, Yeri and Mvolo, and in Lui Hospital, local authorities and healthcare workers considered epilepsy to be one of the major public health problems in the region. Families reported the first cases of NS around 1990. Mvolo, a village located 700 m from the large and fast-flowing Naam River, was reported to be one of the most severely affected villages. We decided to stay for 2 days to investigate the epilepsy situation. Mvolo is infested with blackflies of the *S. damnosum* species complex [11]. The Naam River is a focal point for communities, where men fish, children swim, women wash clothes, and cows graze along the riverbanks. The river becomes very crowded during the fishing season.

The burden of epilepsy was obvious when visiting households in the village. Many children had signs of burns or other injuries resulting from epileptic seizures. A survey of 22 households randomly chosen in two different parts of Mvolo revealed that epilepsy affected 28 out of 168 (16.7 %) children. Thirteen (59 %) households had at least one child with NS or another form of epilepsy, and nine (41 %) had at least two children with NS or another form of epilepsy. Different types of epilepsy (NS, and also convulsive epilepsy not meeting the WHO NS case definition) were often reported in the same family. Generalized epileptic seizures and cognitive deterioration were reported in most children, and several presented with stunted growth and lack of secondary sexual characteristics. The overall epilepsy prevalence was estimated to be 9.2 % (28/306) in Mvolo, and this observation is similar to a prevalence estimate for Mvolo County of 8.4 % (4025/48,100), made by the South Sudan Relief and Rehabilitation Commission (A. Amba, personal communication). These prevalence figures are twice as high as the February 2001 WHO estimates of 4.6 % [3]. There was a common belief among many villagers that epilepsy is transmitted from one child to another. Children with epilepsy were often separated from other children and some were even abandoned. During the 1-week visit to the affected area, villagers reported that two young girls had drowned as the result of seizures while washing clothes in the river.

The distribution of annual doses of ivermectin to residents of Mvolo started in 1996, but the treatment was interrupted several times during periods of conflict (S. Komyangi, personal communication). Recent ivermectin coverage is very low and many new cases of epilepsy continued to appear in Mvolo (J. Podi, May 2015 personal communication). Moreover, high rates of seizures and head nodding were also reported from another onchocerciasis endemic region of South Sudan, in Raga County, Western Bahr El Ghazal. (Y. Biajo, August 2015 personal communication). In South Sudan, very few patients with epilepsy currently have access to anti-epileptic treatment.

Parts of South Sudan have been hyperendemic for human onchocerciasis since at least 1930, and local missionaries reported many combined cases of blindness and epilepsy in the 1950s [11]. Other *Onchocerca* spp. (*O. armillata, O. fasciata* and *O. gutturosa*) have also been reported in domestic animals from the same area [12, 13]. These filarial species can potentially be found in human-biting blackflies (*S. adersi, S. griseicolle* and *S. bovis*) [13].

### Uganda

The epidemic of NS and epilepsy in northern Uganda started during the war between the Ugandan People’s Defence Force (UPDF) and the Lord’s Resistance Army (LRA). The earliest reported onset of NS was in 1997, with the majority of cases occurring beyond 2000, during which time up to 90 % of the war-affected population were forced into internally displaced persons’ (IDP) camps [6]. Many IDP camps were situated close to the fast-flowing Achwa and Pager rivers which were infested with blackflies. Ivermectin was not distributed during the conflict and the extent of onchocerciasis was not known [14, 15]. This probably resulted in high *O. volvulus* infection rates in children with weakened immune systems caused by malnutrition and co-infections. Mass drug administration (MDA) with ivermectin only commenced in Kitgum and Pader districts in 2008. Both Kitgum and Pader were severely affected by NS. Meanwhile, in nearby Adjumani district which has been under MDA for over 15 years, no NS cases have been reported.

The reported clinical manifestations associated with NS in South Sudan and northern Uganda were found to be very similar. In Uganda, management guidelines for the treatment and care of patients with NS were developed in 2012 [16, 17]. Treatment centers are present in all affected districts and are staffed by multi-disciplinary teams of healthcare workers. They provided not only anti-epileptic treatment, but also support for behavioral and emotional difficulties, nutritional therapy, and physical and cognitive rehabilitation. Sodium valproate is the first-line anticonvulsant administered to NS/epilepsy patients [17]. Treatment center teams are supported by village-based lay health volunteers who provide ambulatory care in homes of patients with NS [17]. An evaluation of this program showed that substantial clinical and functional improvements can be obtained with symptomatic treatment, suggesting that NS is not a progressive, debilitating disease, and could even be a reversible encephalopathy in some patients [17].

Whereas the NS epidemic is still ongoing in South Sudan, it appears to have abated in northern Uganda [18, 19]. Uganda is one of three countries in Africa that combine MDA of ivermectin with vector control in order to control onchocerciasis (the other countries being Tanzania and Equatorial Guinea) [20]. MDA of ivermectin was introduced to NS-affected districts of northern Uganda in 2008, and distribution has been biannual since March 2012. Ivermectin MDA was supported by vector control interventions, which began with the aerial application of insecticides to *S. damnosum* breeding-sites along the Achwa and Pager rivers in late-2012. Aerial spraying has since been followed by regular application of larvicides (temephos) to rivers during rainy seasons. Incidence of NS cases has declined in Pader district since 2008, in Lamwo since 2010, and in Kitgum since 2011, with no new NS cases officially reported in Uganda in 2013 [18].

*Simulium damnosum* s.str. is the likely vector of *O. volvulus* in northern Uganda [21], although a recent (unpublished) survey of biting flies indicates that a member of the *Simulium bovis* group accounts for approximately half of the human-biting flies within the NS-affected districts of Kitgum and Lamwo [4].

### Democratic Republic of the Congo

In Liguga, 31 randomly chosen families were interviewed. The population of Liguga health area is 5643, while the town has an estimated 744 inhabitants (Census Community distributor, 2013). Of the 161 children (<18 years old) in the 31 families, five (3.1 %) suffered from epilepsy. Fifty percent of the families reported that at least on child in the family died; 7.9 % of the deaths were caused by epilepsy (epilepsy attack or by drowning).

The majority of epilepsy cases in Liguga, Dingila and Titule presented with generalized (tonic–clonic) seizures without nodding, but with mental retardation. In Titule, in a house to house survey, the prevalence of epilepsy was 2.3 % and patients with epilepsy were clustered in families generally living very close to the rapid-flowing Bima River, which is infested with blackflies [22]. In the three towns, no patients meeting the strict WHO NS case definition were observed, but some children with epilepsy presented with stunted growth and lack of secondary sexual characteristics. A case control study was performed in Titule, details of which will be reported elsewhere. Food was not reported to be a major triggering factor of seizures in Titule, in contrast to what has been reported in South Sudan and northern Uganda [1]. Twenty-seven patients with epilepsy were monitored following consumption of local foods, but none developed head nodding or epileptic seizures. Most children with epilepsy had dropped out of primary school very early because of learning difficulties and stigma associated with epilepsy.

Phenobarbital was available in pharmacies of the villages visited. However, in Titule, 33 (58 %) of 57 patients with generalized convulsive epilepsy had never taken any anti-epileptic medication. In Titule, ivermectin has been distributed annually since 2002. The coverage during the first year of ivermectin distribution was reported to be 23 % (F. Tepage, personal communication). From June 2004 to 2005 ivermectin distribution was temporarily stopped because of reported deaths associated with ivermectin use in *Loa Loa* co-infected patients in the Bas-Congo [23]. Later MDA of ivermectin was restarted and the coverage increased over the years in Titule to 73–81 %. However, of 142 patients with epilepsy interviewed in Titule, 105 (73.9 %) had not taken ivermectin prior to the onset of epilepsy.

In Dingila, a family comprising a husband, two wives and 22 children was interviewed. The husband had seven children with his first wife, four of whom were aged 9, 12, 18 and 20 years and had epilepsy. He had 15 children with his second wife, two of whom were aged 14 and 15 years and had epilepsy. The father used to be a fisherman who had lived for many years in Gbango (N3.71321, E26.00938), a small settlement on the bank of the Uélé River. All his children with epilepsy had lived in Gbango since birth. The fisherman decided to abandon the settlement and moved 12 km away to Dingila because he believed the epilepsy in his children was somehow associated with the river and witchcraft. None of the 15 children born and raised in Dingila have so far developed epilepsy.

*Simulium damnosum* complex blackflies were the dominant species breeding along the main rivers near both surveyed villages and are the principal vectors of *O. volvulus*. While infection rate of the specimens collected during this study is yet to be determined, dissections of adult flies, caught in Titule in 1941, had revealed an infection rate of *O. volvulus* of 18 % [24]. Historical records also mention the presence of human-biting members of the *S. neavei* group in the same region. *Simulium albivirgulatum*, which has potential to bite humans, is also present in the DRC including Bas-Uélé district [25]. Both *S. neavei* and *S. albivirgulatum* are capable of transmitting *O. volvulus*. However, during the survey in Titule and Dingila, the only anthropophilic species collected belonged to the *S. damnosum* complex. Three ornithophilic species were collected in tributaries of both the Bima and Bas-Uélé rivers, namely: *S. cervicornutum, S. unicornutum* and *S. schoutedeni* (identified by R. Post, personal communication). Table 1 summarizes the current status of epilepsy in the three countries of interest.

**Table 1:** State of epilepsy in onchocerciasis endemic regions visited

	Northern Uganda: Gulu, Kitgum, Pader and Lamwo districts	South Sudan: Western Equatoria State	Democratic Republic of the Congo: Bas-Uélé, Orientale Province
Year of first NS cases	NS cases reported since 2000 [1]	NS cases reported since 1990 [2, 3]	Unknown
Tribes	Acholi	Moro and dinka	Bowa, Zande, Lokele, Kango and others
Migration	IDP camps during civil war	IDP camps and “hiding in the forest” during civil war	No important migration, but some “hiding in the forest” during conflicts
Prevalence of all forms of epilepsy	2.9 % (Moyo, Adjumani, Kitgum and Gulu districts, 2010)^a^	9 % (Mvolo, 2013)	2.3 % (Titule, 2014) [22]
Prevalence of NS	0.68 % probable NS cases among children aged 5–18 years in three districts, 2012–2013 [27]	Exact prevalence never assessed but high based on high epilepsy prevalence	No confirmed NS according to WHO case definition, but NS-like suspected cases reported in the region
Stunted growth with lack of secondary sexual characteristics	Present, exact prevalence not reported		Present, not frequent
Nutritional status	Often poor [6]	Often poor [3]	Generally good
Availability of anti- epileptic drugs	Sodium valproate, carbamazepine and phenytoin	Generally not available	Phenobarbital available but often not affordable
Epidemiological situation of NS/epilepsy	NS/epilepsy epidemic until 2013 [18]	Ongoing NS/epilepsy epidemic	Endemic epilepsy
Incidence of NS/epilepsy	Very limited new cases of NS [14]	Still new NS/epilepsy cases	Stable incidence of new epilepsy cases
Ivermectin distribution	Twice a year Not distributed during war	Once a year, low coverage Not distributed during war	Once a year Interrupted in 2004 [23], not distributed during war
Prevalence of onchocerciasis	Decreasing	Not known	Decreasing
Loa Loa endemic region	No	No	Yes
Insecticide/larvicide use	Before 1972 and since 2012 [26]	Only before 1972	Never
General ecology	Savannah forest		Tropical forest
Location of villages	Close to rapid flowing blackfly-breeding rivers		

*NS* nodding syndrome, *IDP* internally displaced persons

^a^ JK personnal communication, unpublished

## Discussion

Observations in South Sudan suggest that there is an ongoing NS and epilepsy epidemic in the Western Equatoria state, while in northern Uganda the epidemic appears to have abated. No new NS cases were officially reported in Uganda in 2013 [18], and only two new NS cases have been reported since then (B. Opar, July 2015 personal communication).

However, our findings should be interpreted with caution. Indeed, during our short site visits, we were unable to perform in-depth and well-designed epidemiological investigations. However, based on our rapid assessment of the epilepsy situation in the three countries, it is clear that there is an urgent need to better quantify and monitor the burden of epilepsy in onchocerciasis endemic regions.

Nodding syndrome and epilepsy epidemics in both South Sudan and northern Uganda occurred in regions hyperendemic for onchocerciasis. In each country, the epidemic occurred at a time where the population lacked access to ivermectin treatment due to disruption in infrastructure caused by civil conflicts [26]. Population displacement, and the location of internally displaced person (IDP) camps close to blackfly infested rivers, where the population had limited access to water from boreholes, may have contributed to these epidemics. Moreover, the lack of epilepsy treatment during the war and in the post-war period may explain the severe forms of cognitive impairment observed in children with NS in both countries.

Whether the distribution of ivermectin and/or blackfly control interventions resulted in a decrease in NS cases in northern Uganda remains to be proven. The use of a stricter NS case-definition in recent years [27] cannot explain the dramatic decrease of NS cases post 2013. However, the already decreasing incidence of NS cases prior to the bi-annual distribution of ivermectin and larviciding of rivers in 2012 could be a result of the annual ivermectin treatment regimen already in place.

In the Bas-Uélé district of DRC, epilepsy also appears to be an important public health problem. Prevalence studies in other districts of the Orientale Province are now also being conducted by the University of Kisangani in collaboration with the Ministry of Health in order to determine the magnitude of the problem. Preliminary data show a prevalence of epilepsy between 2 and 6 % in many villages in the province located near rapid flowing rivers (A. Laudisoit unpublished results). Not all epilepsy in the region will be associated with onchocerciasis, and cysticercosis needs to be excluded as a possible cause, especially given the high number of pigs kept domestically. In Dingila, however, Dr. J.L. Lumaliza collected blood on filter paper from 40 individuals with epilepsy, yet none revealed the presence of cysticercosis antibodies when analysed by enzyme-linked immunoelectrotransfer blot (S. Gabriel unpublished results).

Cases meeting the WHO-approved definition of NS were not observed in Liguga, Dingila, and Titule, but NS-like manifestations were observed (epilepsy cases with stunted growth and lack of secondary sexual characteristics). Suspected cases of NS have also previously been seen by one of us (M. Mandro, unpublished report) in the Logo health zone in Ituri, Orientale Province, DRC.

NS seems to be one of many different types of epilepsy present in onchocerciasis endemic regions. In these regions, different types of epilepsy, including NS and convulsive epilepsy that do not meet the WHO case definition of NS occur among children within the same family, suggesting that different clinical presentations of epilepsy may be caused by a common trigger the family is exposed to.

By strictly adhering to the WHO case definition of NS, we may significantly underestimate the burden of the disease. A recent study to estimate the prevalence of NS in three districts of northern Uganda highlighted the difficulty in applying the WHO NS case definition [27].

Epidemic epilepsy of unknown etiology, clustered in onchocerciasis areas, has previously been referred to as “river epilepsy” by French researchers working in the Mbam valley, an onchocerciasis endemic area in Cameroon [8, 28]. A similar type of epilepsy seems to be present in the DRC. The presence in the DRC of epilepsy with an onset of during childhood in older people suggests that it is not necessarily a progressive degenerative disease and that it has been endemic in the Province Orientale of the DRC for a long time. Recent studies in Tanzania and Uganda also suggest that NS is not necessarily a progressive disease [16, 29, 30]. Most children with NS who receive adequate care, including anti-epileptic treatment, nutritional and emotional support, and physical rehabilitation, improve clinically. A reduced frequency of seizures and even reversal of severe functional disabilities have been reported, including by the NS treatment center “Hope for Humans”, which we visited in Odek [16, 29]. There is an urgent need for the government of South Sudan to replicate the Ugandan NS care and treatment program in their affected villages, to provide funding and seek relevant expertise in order to implement such a program.

Living in close proximity to rapid-flowing, blackfly-infested rivers, and in areas of low ivermectin coverage, appear to be the common factors in the three regions of South Sudan, northern Uganda and DRC with a high prevalence of epilepsy. Blackflies may play a key role in transmitting an etiological agent that directly or indirectly is causing epilepsy. Our preliminary observations support the need to conduct fundamental and systematic research on *Onchocerca* spp., their hosts, and vectors in these regions. For example, the possible occurrence of novel strains of *Onchocerca* and associated *Wolbachia* in areas of high epilepsy prevalence should be investigated. It is already suspected, but not confirmed, that different *O. volvulus* strains exist that are responsible for differences observed in clinical disease (blinding/skin lesions) [31].

A recent study suggests that an antibody-mediated autoimmune response to leiomodin-1 is involved in the etiology of NS, and that autoimmunity due to molecular mimicry of an *O. volvulus* surface protein contributes to NS pathogenesis [32]. The authors showed that 11 (58 %) of 19 patients with NS had detectable serum autoantibodies to leiomodin-1, compared to five (26 %) of 19 controls (matched OR 7.0, 95 % CI 0.9–11.1). These antibodies, which are also present in the CSF of patients with NS, were found to be neurotoxic in vitro and cross-reacted with *O. volvulus* tropomyosin which has regions of significant homology with leiomodin-1. While it is hard to envisage that the target of functional antibodies against *O. volvulus* is a muscle isoform of tropomyosin (which is only exposed when microfilariae are dead), it seems possible that the muscle isoform from dying or dead microfilariae induces a humoral response that gives rise to cross-reactivity with a non-muscle form, leading to active parasite destruction most likely through an antibody-dependent cell-mediated cytotoxicity reaction [33]. It has been suggested that the pathogenesis of retinochoroidal changes in onchocerciasis is also due to an autoimmune reaction involving cross-reacting antigens between *O. volvulus* and retinal components [34–38]. In a recent study in Uganda, serum antibodies against voltage gated potassium channel (VGKC)-complex proteins were detected in 15 (48 %) of 31 patients with NS compared to only in 1 (9 %) of 11 sibling controls (R. Idro, unpublished). Such antibodies were previously not detected among patients with NS in Tanzania [5] but there have been concerns about the methodology that was used to detect these antibodies in the Tanzanian study.

If we can demonstrate that improving ivermectin coverage can decrease the incidence of onchocerciasis associated epilepsy or “river epilepsy”, this will improve the acceptance of the drug among populations where onchocerciasis is largely asymptomatic, and it would be an additional argument to strengthen onchocerciasis elimination efforts.

The burden of disease caused by epilepsy in onchocerciasis endemic areas is much higher than the 3300 estimated cases of NS in Uganda [27]. Indeed, a high prevalence of epilepsy has been reported in many onchocerciasis endemic areas [7, 8, 39–43]. The prevalence of epilepsy in three African demographic surveillance sites, non-endemic for onchocerciasis (Agincourt, South Africa; Iganga-Mayuge, Uganda; and Kilifi, Kenya), was 2.3 per 1000 (ten times lower than the prevalence in Titule) [44].

NS and “onchocerciasis associated epilepsy” (or “river epilepsy”) remain very neglected public health problems. It was only in April 2014 that was NS added to the WHO list of neglected tropical diseases as “another condition”. In Table 2 we propose a clinical definition of “onchocerciasis associated epilepsy” (or “river epilepsy”) that could be used for surveillance purposes. A multi-country, multidisciplinary research effort will be required to eliminate the suffering caused by this condition. We hope our preliminary data from South Sudan and the DRC not only will motivate funding agencies to support further research on epilepsy in these remote onchocerciasis endemic regions, but above all will motivate them to fund and set up treatment programs for children with epilepsy in these regions.

**Table 2:** Proposed clinical case definition for “onchocerciasis associated epilepsy” (“river epilepsy”)

Major criteria
A previously healthy person in an onchocerciasis endemic area who develops head-nodding or convulsive epilepsy of unknown etiology between the ages 3 and 18 Plus at least one of the following:
Minor criteria
Other neurological abnormalities (cognitive decline, school dropout due to cognitive/behavioral problems, psychiatric manifestations, other neurological abnormalities) Clustering in space or time with similar cases Stunting or wasting Delayed sexual development
